# Work-related problems and the psychosocial characteristics of individuals with irritable bowel syndrome: an updated literature review

**DOI:** 10.1186/s13030-024-00309-5

**Published:** 2024-05-15

**Authors:** Nagisa Sugaya

**Affiliations:** Occupational Stress and Health Management Research Group, National Institute of Occupational Safety and Health, 6-21-1 Nagao, Tama-ku, Kawasaki, Kanagawa 214-8585 Japan

**Keywords:** Irritable bowel syndrome, Workers, Work productivity

## Abstract

**Background:**

Irritable bowel syndrome (IBS) is a common, functional gastrointestinal disorder. Because IBS often develops and worsens with stress, it requires treatment from both physical and mental perspectives. Recent years have seen increasing reports of its impact on the daily performance and productivity of workers with IBS, leading to sick leaves and lower quality of life. Therefore, this narrative review aimed to summarize the work and psychosocial characteristics of individuals with IBS.

**Main body:**

Workers with IBS report greater occupational stressors and work productivity impairments, including presenteeism or absenteeism, in addition to suffering from psychological distress, low quality of life, and medical and economic problems, similar to those with IBS in the general population. Anxiety about abdominal symptoms, as well as the severity of IBS, is related to the degree of interference with one’s work. Regarding the association between characteristics of work and IBS, shift work and job demands/discretion have been associated with IBS. Studies on specific occupations have revealed associations between IBS and various occupational stressors in healthcare workers, firefighters, and military personnel. Telecommuting, which has become increasingly popular during the coronavirus disease pandemic, has not found to improve IBS. Moreover, the effectiveness of medication, diet, and a comprehensive self-management program, including cognitive behavioral therapy, in improving the productivity of workers with IBS have been examined.

**Conclusion:**

As mentioned above, the IBS of workers is related not only to their problematic physical and mental health but also to work-related problems; workers with IBS exhibit severe occupational stress factors and work productivity impairment. Further research is required to develop efficient and appropriate interventions for workers.

## Background

Irritable bowel syndrome (IBS) is a common, functional gastrointestinal disorder. The Rome IV criteria [1], the latest international diagnostic criteria, define IBS as recurrent abdominal pain occurring on average at least 1 day per week in the last 3 months and associated with at least two of the following three abnormalities: (1) defecation-related, (2) change in defecation frequency, or (3) change in stool characteristics (appearance). Patients were subtyped according to their predominant bowel habits into four subtypes: IBS with diarrhea, IBS with constipation, IBS with mixed bowel habits (IBS-M), and IBS unclassified. The pathophysiology of IBS [2] includes (1) abnormalities in gastrointestinal motility, (2) decreased gastrointestinal sensory threshold, and (3) psychological abnormalities (anxiety, depression, etc.), all of which are related to abnormalities in the functional relation between the brain and intestine (the brain-gut interaction). Prevalence rates in the general population obtained from online surveys are relatively high: approximately 4% based on the latest diagnostic criteria, Rome IV, and 10% based on the previous criteria, Rome III [3]. Because IBS is a gastrointestinal psychosomatic disorder that often develops and worsens with stress, it requires treatment from both a physical and mental perspective [4].

IBS has been associated with medical and economic problems [5], and its overall economic impact is likely to be even higher because of its physical and psychological effects on work capacity. IBS is particularly prevalent among prime-age workers, and recent years have seen increasing reports of its impact on the daily performance and labor productivity of workers with IBS, leading to sick leaves and a lower quality of life [6]. Therefore, this narrative review aimed to summarize the work and psychosocial characteristics of individuals with IBS and to contribute to future observational and interventional research that will lead to effective strategies for workers with IBS.

## Methods

Major literature databases, including PubMed, APA PsycInfo, and Google Scholar, were searched for suitable articles, including all publication years up to December 2023. The search strategy included the following terms: (irritable bowel syndrome OR IBS) AND (work OR employ OR occupation). The abstracts and titles of relevant articles were screened and only original papers and meta-analyses written in English were included in this review. Additional studies were identified from the reference lists of the screened articles. The included studies were summarized in terms of (1) the psychosocial characteristics of workers with IBS, (2) the association between features of work and IBS, (3) the effect of IBS on work productivity, and (4) the effect of treatments for IBS on work.

## Psychosocial characteristics of workers with IBS

Many studies have revealed an association between IBS and low quality of life [7–9]. A study of healthcare workers in Italy reported a higher prevalence of mental (anxiety and mood disorders) and sleep disorders in individuals with IBS than in those without IBS [10]. A Korean study reported that female nurses with IBS were approximately two times more likely to experience depressive symptoms than those without IBS when various confounding variables were considered [11]. A study of resident doctors in China also showed that individuals with IBS had more severe depression and perceived stress [12]. Moreover, workers with IBS in Israel experience higher burnout than those without IBS [13]. However, this result is not characteristically specific to workers but is common to IBS in general. Regarding worker-specific characteristics, a study in Romania [14] reported higher occupational stress and interleukin-6 (an inflammatory cytokine) levels in workers with IBS than in those without, and these indices were correlated.

While medical-economic problems associated with IBS occur in the general population, not just in the workforce [5], indirect costs, including work productivity impairment, account for a large portion of the overall economic burden of IBS. Medical and economic studies of workers have been conducted for a long time. A study conducted in the United States more than 20 years ago that measured medical and pharmaceutical (direct costs) and disability and medically related workplace absenteeism costs (indirect costs) associated with IBS to determine the overall economic burden of IBS on an employer revealed a significant economic burden on employers arising from increased direct and indirect costs related to workers with IBS [15]. A recent study in the United Kingdom reported that clusters with a higher psychological burden among individuals with IBS had higher IBS-related medical costs in the past 12 months as well as greater impairment in productivity and ability to work [16].

## Features of work and IBS

Research has revealed a relation between IBS and various features of work. In a study in Saudi Arabia, freelance employees who worked for more than nine hours were more likely to have IBS [7]. One meta-analysis reported that shift work is associated with IBS [17], and another meta-analysis of healthcare professionals found that in addition to shift work, poor sleep quality and female sex were also major contributors to the presence of IBS [18]. Shift work may also contribute to circadian disturbances. Gastrointestinal activity is regulated by a circadian rhythm of 24 h per day. Circadian rhythm disturbances may alter the intestinal microbiota and regulate gastrointestinal motility, secretion, immunity, and metabolism [19, 20]. This biological background may mediate the association between shift work and IBS.

Several studies have reported on the impact of job demands and discretion on IBS. In a Chilean study, although high job demands doubled the prevalence of IBS, high job skill discretion appeared to reduce the prevalence of IBS in the presence of high job demands [21]. In a study that also examined patients by sex in the United States, male patients with IBS reported fewer decisions about the pace of work compared to those reported by non-IBS individuals, and female patients with IBS reported less decision-making authority about work plans and fewer opportunities for learning in the workplace [22]. These relationships have been studied using the job-strain model [23], and high demands combined with low control at work has been found to be related to an increased risk of stress-related diseases [24]. Based on this model and previous research, the degree of control and work demands can be interpreted as influencing IBS symptoms. However, as the above study [22] discussed, reverse causality is also possible, and IBS symptoms may cause a selection process in which patients with IBS, predominantly women, more frequently get low-demand jobs where their decision-making authority is also low.

Occupation-specific studies have also been conducted. An Italian study on health workers found that lower levels of social support, control at work, work capacity, job demands, work strain, and “isostrain” (stress with lack of support and isolation; in this study, the isostrain score was stress divided by support) correlated with the severity of IBS [10]. A study on firefighters in South Korea revealed a higher IBS risk in the following occupational stress subcategories: job demand, interpersonal conflict, organizational system, and lack of rewards [25]. A study of active-duty non-combat Greek military personnel also suggested that severe anxiety and occupational stress were related to an IBS diagnosis [26]. As described above, there are common aggravating factors of IBS among occupations; however, the points at which an intervention can be made and the environment in which they can be changed may differ, and it is necessary to consider occupation-specific measures.

Research has also revealed the impact of working from home on IBS patients. The coronavirus disease (COVID-19) pandemic has caused economic and psychological damage to people and major social changes, including the widespread use of working from home [27, 28]. While working from home may lead to less interaction and social support, it also increases freedom in terms of one's behavior (e.g., going to the bathroom) in a place where one is unobserved by the surrounding community. Therefore, working from home can have both positive and negative effects on the psychological state of IBS patients. One study showed that among telecommuting IBS patients (*N* = 42), 42.9% had worsening symptoms, 57.1% had unchanging or improving symptoms, and among those with IBS who went to work (*N* = 25), 28% had worsening symptoms and 72% had unchanged or improved symptoms, indicating that telecommuting did not lead to an improvement in symptoms. The results revealed that working from home did not lead to symptom improvement [29]. The authors of this study discuss how multiple factors, including lack of social support, occupational stress, and burnout, may have exacerbated IBS symptoms, counteracting the effect of telecommuting in improving IBS symptoms. However, these results were obtained under the special circumstances of a pandemic and require verification after the pandemic ends. It will be necessary to examine how changes caused by telecommuting, such as freedom of access to toilets, not using public transport, and communication styles with supervisors and colleagues, affects IBS symptoms and various other aspects, including cognitive and behavioral aspects related to IBS. This could contribute to effective work environment adjustments, including telecommuting, for workers with IBS.

## Effect of IBS on work productivity

IBS has been reported to have a significant impact on the loss of work productivity (Table 1). A commonly used instrument for assessing loss of work productivity due to health problems is the Work Productivity and Activity Impairment (WPAI) [30]. Many versions of the WPAI have been developed, with questions modified for different diseases, including a version specific to IBS (WPAI:IBS) [31]. Some studies have used the WPAI:IBS. In a study in the United States, workers with IBS were 15% less productive at work due to gastrointestinal symptoms compared to non-IBS workers, and the presence of IBS was associated with a 21% decrease in work productivity, equivalent to working less than four days out of a 5-day work week [8]. A Canadian study reported that absenteeism (percentage of work time missed) and presenteeism (percentage of impairment while at work) due to IBS symptoms (as assessed by the WPAI:IBS) were 5.6% and 31.4%, respectively [32]. Another study in the United Kingdom found that absenteeism and presenteeism due to IBS symptoms were 28.5% and 85.6%, respectively [6]. One factor may be the differences between the results of the studies by Paré et al. (2006) [32] and Goodoory et al. (2022) [6]. The former study used Rome II and the latter Rome IV: because the use of Rome IV results in the detection of less IBS prevalence than does past IBS diagnostic criteria, the latter study might have included individuals with more severe IBS than the former. A report in Sweden indicated that the severity of IBS symptoms was related to presenteeism [33]. A study on the characteristics of each IBS subtype in the United States reported that individuals with constipation-predominant IBS had higher presenteeism, overall work impairment, and activity impairment than controls [34], and that individuals with diarrhea-predominant IBS had greater absenteeism or presenteeism, lower overall work productivity, and greater activity impairment than the non-IBS group [35]. Furthermore, the presence of IBS has been associated with unemployment in Sweden [36]. These results support the idea that IBS may lead to indirect economic problems. Investigating the mediating factors between IBS and work productivity impairment may contribute to improving both the QOL of workers with IBS and their health economic problems.

**Table 1:** Effect of IBS on work productivity

Author (year)	Country	Diagnostic criteria for IBS	Participants	Main results for work productivity
Goodoory et al. (2022) [6]	United Kingdom	Rome IV	752 individuals with IBS (workers: 62%)	1) Among the employed individuals, 28.5, 85.6, and 81.8% reported absenteeism, presenteeism, and overall work impairment, respectively. 2) Among all participants, 91.0% reported activity impairment (home management: 29.3%, social leisure: 56.3%, private leisure: 27.5%, and maintaining close relationships: 27.0%). 3) Severe IBS, anxiety, depression, somatization, gastrointestinal symptom-specific anxiety, and decreased IBS-related QOL were associated with impairment of work and activities of daily living.
Dean et al. (2005) [8]	United States	Rome II	1,776 employees (IBS: 41%)	1) Employees with IBS had a 15% greater loss in work productivity because of gastrointestinal symptoms and lower health-related QOL than those without IBS. 2) IBS was associated with a 21% reduction in work productivity, equivalent to working < 4 d in a 5-d workweek.
Paré et al. (2006) [32]	Canada	Rome II	1,555 IBS patients (workers: 59.3%)	Patients reported 5.6, 31.4, and 34.6% absenteeism, presenteeism, and overall work productivity impairment, respectively.
Frändemark et al. (2018) [33]	Sweden	Rome III	525 IBS patients (workers: 70%)	1) Of the employed patients, 24.3 and 86.8% reported absenteeism and presenteeism because of IBS, respectively. 2) Gastrointestinal-specific anxiety was associated with absenteeism and overall work loss. 3) IBS severity was associated with presenteeism, overall work loss, and activity impairment.
DiBonaventura et al. (2011) [34]	United States	Rome II	789 patients with IBS-C (workers: 51%) and 789 healthy controls (workers: 56%)	IBS-C patients had a lower health-related QOL score and higher presenteeism, overall work impairment, and activity impairment scores.
Buono et al. (2017) [35]	United States	Rome II	1,102 individuals with IBS-D and 65,389 controls without IBS-D or inflammatory bowel disease (workers: 54%)	1) Individuals with IBS-D had a lower health-related QOL score and higher absenteeism, presenteeism, overall work productivity impairment, and activity impairment scores. 2) Individuals with IBS-D incurred an estimated $2,486 more in indirect costs.
Nilsson et al. (2021) [36]	Sweden	Rome III	2,648 participants (IBS: 11.9%) Not all of them are workers.	1) In men, IBS was associated with middle-age and both IBS and gastrointestinal symptoms were associated with unemployment. 2) In women, IBS was associated with present smoking, and gastrointestinal symptoms were associated with former smoking and inversely associated with higher age and intermediate physical activity at work.
Silk et al. (2001) [38]	United Kingdom	-	1,597 IBS patients (workers: 44%)	1) Overall, 12 % of respondents gave up work altogether because of IBS, and although 47% of employed respondents reported having taken time off work, only 35% of those respondents cited IBS as a reason. 2) Employers who were informed of the diagnosis of IBS accepted this condition as a valid reason for absence from work in 61% of cases. 3) Overall, 53% employees suffered from embarrassment when using the restroom at work, and 32% reported that IBS prevents them from applying for promotions or new jobs.

*IBS* Irritable bowel syndrome, *IBS-C* Constipation-predominant IBS, *IBS-D* Diarrhea-predominant IBS

Various factors may underlie the work-related disruptions of IBS patients. For example, a Swedish study found that gastrointestinal symptom-related anxiety was associated with absenteeism [33]. This study reported that the associations between clinically diagnosed IBS status and work interference due to gastrointestinal symptoms were also observed both directly and indirectly via a sense of coherence (reflecting the degree to which the individual can effectively apply coping strategies to the stresses of daily life) and confidence in the healthcare system, with a sense of coherence appearing to be particularly important [37]. In a United Kingdom study, greater IBS severity, higher gastrointestinal symptom-specific anxiety, and lower IBS-related quality of life were associated with disability in both work and activities of daily living [6], and more than half (53%) of employed individuals with IBS experienced embarrassment in the bathroom at work, with 32% reporting that they were unable to apply for promotions or new jobs because of their IBS [38]. Thus, the association between IBS and impaired work productivity may be mediated by factors traditionally targeted in psychotherapy for IBS, such as abdominal symptom-related anxiety, shame, and stress coping strategies.

As described above, IBS has a significant impact on loss of work productivity, which may be related to how abdominal symptoms are interpreted and what kinds of behavioral responses are generated in response to the symptoms. Further research on how occupational stressors, including the work environment, may interact with the relations between these variables will aid the development of effective psychological interventions for workers with IBS.

## Effect of treatments for IBS on work

Several studies have reported on the effectiveness of various IBS treatments on work productivity (Table 2). Regarding the effectiveness of pharmacological treatment for IBS on work productivity, some randomized controlled clinical trials have reported that tegaserod (5-HT4 receptor agonist) [39] and linaclotide (guanylate cyclase C agonist) [40] improved the work productivity of patients with constipation-predominant IBS. For patients with diarrhea-predominant IBS reporting inadequate response to loperamide (μ-opioid receptor agonist), treatment with eluxadoline (mixed μ-opioid and κ-opioid receptor agonist and δ-opioid receptor antagonist) was associated with significant improvement in absenteeism due to IBS symptoms and health and it reduced presenteeism, overall work productivity loss, and daily activity impairment, although the effect was not statistically significant [41]. For dietary treatment, IBS patients with diarrhea who were randomized to a low-fermentable oligo-, di-, and monosaccharide and polyol (FODMAP) diet showed a greater reduction in activity impairment after a 28-day intervention [42]. A comprehensive self-management program, including cognitive-behavioral therapy, dietary education, and relaxation, was effective in improving presenteeism, overall work productivity, and activity impairment, particularly among those with greater baseline work/activity impairment [43]. These results suggest that improving the abdominal symptoms of IBS and associated psychological problems may increase work productivity, as well as improve quality of life and health related economic problems.

**Table 2:** Effect of IBS treatment on work

Author (year)	Country	Diagnostic criteria of IBS	Study design	Participants	Procedure	Results
Reilly et al. (2005) [39]	United states, 14 Europe countries, 5 South America countries, Canada, Egypt, New Zealand, and South Africa	Rome II	A prospective, randomized, double-blind, placebo-controlled trial	1,675 female patients with IBS-C (All were employed.)	Participants were assigned to a trial of tegaserod 6 mg b.d. or placebo. IBS-related work productivity and activity impairment was assessed at baseline and weeks 2 and 4.	1) Compared with placebo, tegaserod reduced work and daily activity impairment at weeks 2 and 4. 2) Tegaserod reduced absenteeism, presenteeism, overall work productivity loss, and activity impairment at week 4 compared with baseline.
Buono et al. (2014) [40]	United States	Rome II	Two prospective, randomized, double-blind, placebo-controlled trials	1555 IBS-C patients (71.7% of them were employed.)	Participants were assigned to the trial of an oral capsule of linaclotide 290 μg once daily or placebo. Assessments of IBS-C-related work productivity/activity impairment were conducted at baseline and at weeks 4, 8, and 12 during the 12-week treatment periods in Trials 1 and 2 and at weeks 16, 20, and 26 during the extended treatment period in Trial 2.	1) Linaclotide therapy significantly reduced overall work productivity impairment and daily activity impairment among patients with IBS-C at all study weeks. 2) The reduction in overall work productivity impairment from baseline to the 26th week was equivalent to 103 to 156 hours per year, or $3,209 to $4,861 per year in overall labor loss avoided for IBS-C employees.
Brenner (2021) [41]	United States	Rome III	A prospective, randomized, double-blind, placebo-controlled trial	346 patients with IBS-D who reported previous inadequate response to loperamide. (41.9% were employed.)	Participants were administered oral eluxadoline (100 mg) or placebo twice daily for 12 weeks. Health outcome assessments, including IBS-D-related work productivity and health-related QOL were administered to patients at baseline, week 4, week 8, and at the end of the 12-week treatment period.	1) In comparison with placebo from baseline to week 12, eluxadoline treatment resulted in a significantly greater reduction in absenteeism. 2) From baseline to week 12, eluxadoline treatment led to a significantly greater reduction in the number of unhealthy days experienced.
Eswaran et al. (2017) [42]	United States	Rome III	A prospective, randomized, single-blind, controlled trial	92 adult patients with IBS-D (64.2% of them were employed.)	Participants were assigned to groups placed on a 4-week diet low in FODMAPs or a modified diet recommended by the mNICE. IBS-related QOL, psychosocial distress, work productivity, and sleep quality were assessed before and after the diet period.	1) At 4 weeks, patients on the diet low in FODMAPs had a larger mean increase in IBS-related QOL than did patients on the mNICE diet. 2) Anxiety decreased in the low-FODMAP diet group compared with the mNICE group. 3) Activity impairment was reduced with the low-FODMAP diet compared with the mNICE diet.
Yang et al. (2022) [43]	United States	Rome II	A prospective, randomized, single-blind, controlled trial	160 IBS patients (Workers or students were 79.7% of the participants.)	Participants were divided into 2 groups; one with a comprehensive self-management (CSM) intervention program (including CBT, dietary education, and relaxation) for work and activity impairment and a usual care group. Data for IBS-related work productivity and activity impairment collected at baseline and 3-, 6- and 12-months post-randomization were analyzed.	1) The effect of CSM was shown to be superior to usual care in improving IBS-related work productivity and activity, with sustained effects up to 12 months post-randomization. 2) The CSM intervention was found to be particularly beneficial for IBS patients with greater baseline work and activity impairments.

*IBS* Irritable bowel syndrome, *IBS-C* Constipation-predominant IBS, *IBS-D* Diarrhea-predominant IBS, *FODMAP* Fermentable oligo-, di-, and monosaccharides and polyols, *mNICE* National Institute for Health and Care Excellence, *CBT* Cognitive behavioral therapy, *CSM* Comprehensive self-management

Recently, various interventions via the internet and other means have been proven effective for individuals with IBS. Internet-delivered cognitive behavioral therapy could be a cost-effective intervention to improve the symptoms and quality of life in patients with IBS [44]. Additionally, artificial intelligence dietary mobile apps have improved IBS-related QOL and bowel habit satisfaction [45]. Therefore, conducting these interventions via the internet as well as at medical institutions may facilitate access to appropriate and cost-effective treatments, especially when IBS patients cannot attend medical facilities in person.

## Conclusion

The IBS of workers is associated with not only their problematic physical and mental health but also work-related problems. Workers with IBS exhibit severe occupational stress and work productivity impairment, including presenteeism and absenteeism. Further research on the associations among cognitive-behavioral problems caused by IBS, job stressors (e.g., job overload, discretionary authority over work), and work productivity will be necessary to develop efficient and appropriate interventions for workers. Another potential novel topic is identifying the factors that influence the impact of telecommuting on IBS, a work style that has rapidly become popular in recent years. In addition, by examining the psychosocial factors that individuals with IBS need to improve in order to continue working, the consideration of the characteristics of their occupation and workplace would provide more practical and useful information.

## Data Availability

Not applicable.

## Abbreviations

IBS: Irritable bowel syndrome
IBS-M: IBS with mixed bowel habits
WPAI: Work Productivity and Activity Impairment
IBS-C: Constipation-predominant IBS
IBS-D: Diarrhea-predominant IBS
FODMAP: Fermentable oligo-, di-, and monosaccharides and polyols
mNICE: National Institute for Health and Care Excellence
CBT: Cognitive behavioral therapy
CSM: Comprehensive self-management
